# The construction of intrahepatic cholangiocarcinoma model in zebrafish

**DOI:** 10.1038/s41598-017-13815-0

**Published:** 2017-10-17

**Authors:** Jing Wang, Xiaoqian Leng, Guiping Wang, Xiaoyang Wan, Hong Cao

**Affiliations:** 1 State Key Laboratory of Freshwater Ecology and Biotechnology, Institute of Hydrobiology, Chinese Academy of Sciences, Wuhan, 430072 China; 2 Key Laboratory of Freshwater Biodiversity Conservation, Ministry of Agriculture of China, Yangtze River Fisheries Research Institute, Chinese Academy of Fisheries Science, Wuhan, 430223 China

## Abstract

Intrahepatic cholangiocarcinoma (ICC) is a highly malignant tumor, difficult to diagnose even at an early stage. In this study, we successfully constructed an *nras* ^*61K*^-induced ICC model in zebrafish. Transcriptome analysis and gene set enrichment analysis (GSEA) of liver samples of the ICC and WT (wild-type) zebrafish revealed that the genes differentially expressed between the two groups were mainly involved in focal adhesion, chemokine signaling and metabolic pathways. Analysis of DNA methylomes revealed that compared with WT samples, methylated genes in ICC samples were enriched in functions associated with cellular, single-organism and metabolic processes. In particular, our result discovered eleven potential biomarker genes of ICC which were conserved between zebrafish and humans. Moreover, three potential biomarker genes were hypomethylated in the tumorigenesis of ICC: *ehf*, *epha4* and *itgb6*. In summary, our study provides a comprehensive analysis of molecular mechanisms accompanying the progressive *nras* ^*61K*^-induced ICC. This work indicates that our transgenic zebrafish could be a valuable model, not only for studying liver cancer, but also for exploring new therapeutic targets.

## Introduction

Liver cancer is an umbrella term for many cancer subtypes, including the intrahepatic cholangiocarcinoma (ICC), hepatocellular carcinoma (HCC), hepatoblastoma (HB), fibrolamellar hepatocellular carcinoma (FL-HCC), etc. The ICC is the second most common liver cancer worldwide^1^, highly malignant and difficult to diagnose even at an early stage. Thus, it is very important to construct an ICC animal model and identify disease-specific biomarkers. With the advances in transgenic techniques in the past few decades, zebrafish has become a high-through put and cost-effective experimental model for cancer research. This includes lymphoblastic T-cell leukemia^2^, melanoma^3^, rhabdomyosarcoma^4^ and pancreatic cancer^5^. Since liver cancer is one of the most deadly cancers, several molecular studies have used zebrafish as the model for this cancer. In recent years, Gong’s group generated inducible *kras* ^*V12*^ transgenic^6^ and Xmrk-induced HCC model to study the liver tumorigenesis^7^. Another model developed by Wu’s laboratory conditionally coexpressed hepatitis B virus X (HBx) and hepatitis C virus core (HCP) in zebrafish livers, which caused the formation of ICC^8^.

The RAS proteins control several signal transduction pathways involved in the normal cell growth and malignant transformation^9^. Aberrant activation of the RAS pathway is ubiquitous in most human tumours, whether due to mutations of the RAS genes themselves or to alterations in the upstream or downstream signalling components, such as the Raf-MEK-ERK and PI3K-AKT-mTOR pathways^10–12^. Indeed, some on-going rational therapies targeting RAS and its downstream signaling cascades in HCC could inhibit tumour growth, survival and spread. Sorafenib, a multi-target-directed drug that blocks Ras-Raf-MEK-ERK and VEGF pathways is now approved for the treatment in HCC^13,14^. This demonstrates the potential of RAS signaling as an attractive target for pharmacological design in the treatment of liver cancer. In human liver cancers, approximately 7% and 4% cases carry activating mutations in the *kras* and *nras* oncogenes, respectively^9^. Although the transgenic *kras* ^*V12*^-induced HCC model was reported before^6^, and revealed several molecular mechanisms underlying *kras* driven liver tumorigenesis, as well as recapitulated the typical hallmarks of human HCC, the pathological mechanisms of *nras* mutations were less known.

DNA methylation is an indispensable epigenetic regulatory mechanism. Aberrations in DNA methylation often lead to harmful alterations in gene expression, which is a major hallmark of tumor progression^15–19^. For example, hypermethylation in promoter regions of tumor suppressor genes often results in transcriptional silencing of those genes, which then drives the cancer initiation^20^. In addition, DNA methylation changes in other gene body regions in oncogenes may also contribute to the pathogenesis of cancer^21^. Abnormal DNA methylation frequently appears in various cancers. With regards to the liver cancer research, studies revealed massive epigenetic alternations in HCC, indicating that deregulation of DNA methylation plays an important role in tumorigenesis and metastasis^22,23^.

In this study, we successfully constructed an ICC model in zebrafish using the liver-specific *fabp10* (fatty acid binding protein 10) promoter to overexpress oncogenic *nras* ^*61K*^ specifically in the transgenic zebrafish liver, evidenced by histological diagnosis. Moreover, we utilized this zebrafish ICC model to investigate genome-wide DNA methylomes in reference to normal zebrafish. Our study provided a comprehensive analysis of molecular mechanisms during the progressive *nras* ^*61K*^-induced ICC. This work indicates that our transgenic zebrafish could be a valuable model, not only for studying liver cancer but also for exploring new therapeutic targets.

## Results

### Generation of *Tg*(*fabp10:nras*^*61K*^) transgenic zebrafish

We constructed *Tg*(*fabp10:nras* ^*61K*^) transgenic zebrafish, in which the gene expression was driven under the control of the liver-specific *fabp10* promoter (Supplementary Fig. 1A). To verify if *Tg*(*fabp10:nras* ^*61K*^) transgenic zebrafish was successful, we examined the fluorescence of two-day-old larvae by inspecting the green fluorescence of the GFP gene in the livers of F_0_ fish. Western blot analyses confirmed that the fusion protein Nras^61K^-GFP could be detected in the ICC samples, but not WT (Supplementary Fig. 1B,C). Two founders transmitted the transgene to their progenies (F_1_) and carried the transgene insertions in germ cells. In addition, to identify the integration locus, we sequenced the 5′ flanking fragment of expression cassettes in two lines. The integration site of line 1 was idntified at the loci 27630228 to 27631132 of the chromosome 15, while the integration site of line 2 was idntified at the loci 55341075 to 55341374 of the chromosome 20. Both of the two integration loci of cassettes are not tagged genes or introns of genes.

### Induction of ICC in the livers of *Tg*(*fabp10:nras*^*61K*^) transgenic zebrafish

**Figure 1:**
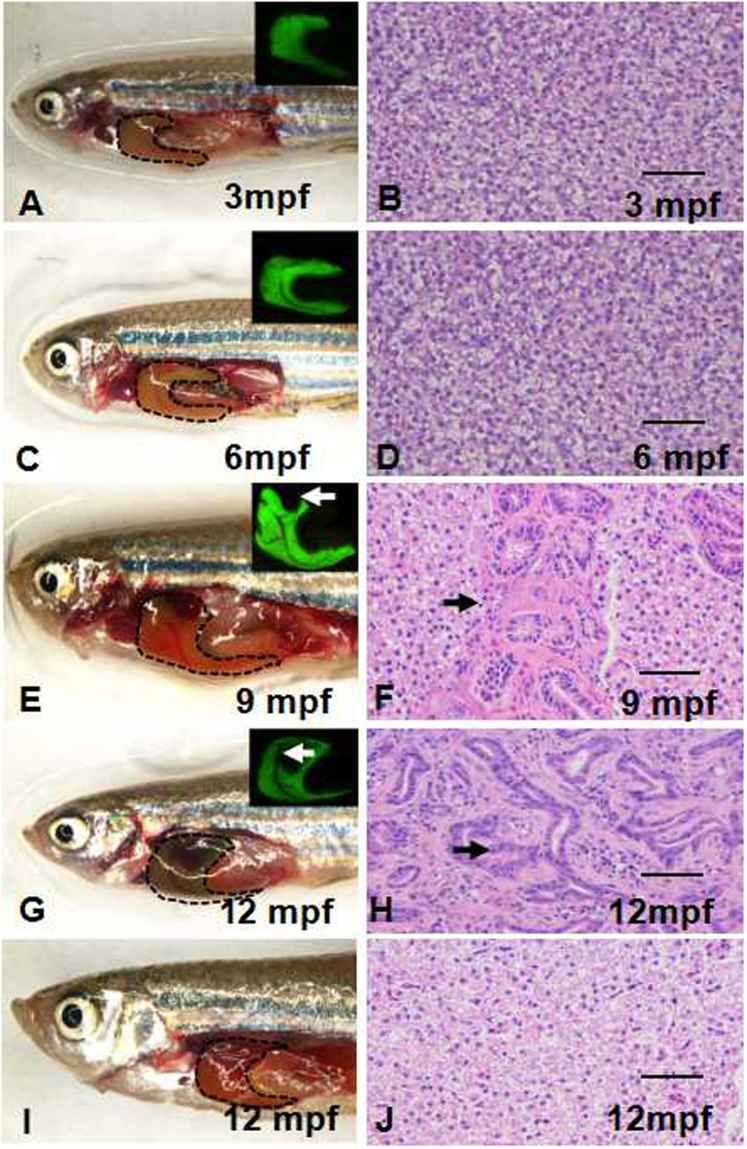
The liver tumor in *Tg*(*fabp10:nras* ^*61K*^) transgenic zebrafish. The fluorescence of EGFP was used to detect the *nras* ^*61K*^ gene expression. (**A**,**C**,**E**,**G**) Brightfield and fluorescence (insets) images displaying the gross morphology of F_4_ *Tg*(*fabp10:nras* ^*61K*^) transgenic zebrafish at 3, 6, 9 and 12 mpf. Tumor protrusions are indicated by arrowheads. (**B**,**D**,**F**,**H**) Histology of F_4_ *Tg*(*fabp10:nras* ^*61K*^) transgenic zebrafish at 3, 6, 9 and 12 mpf. (**I**) Brightfield images displaying the gross morphology of WT zebrafish at 12 mpf. (**J**) Histology of WT zebrafish at 12 mpf. Dotted areas indicate liver regions. Scale bars: 50 µm.

**Figure 2:**
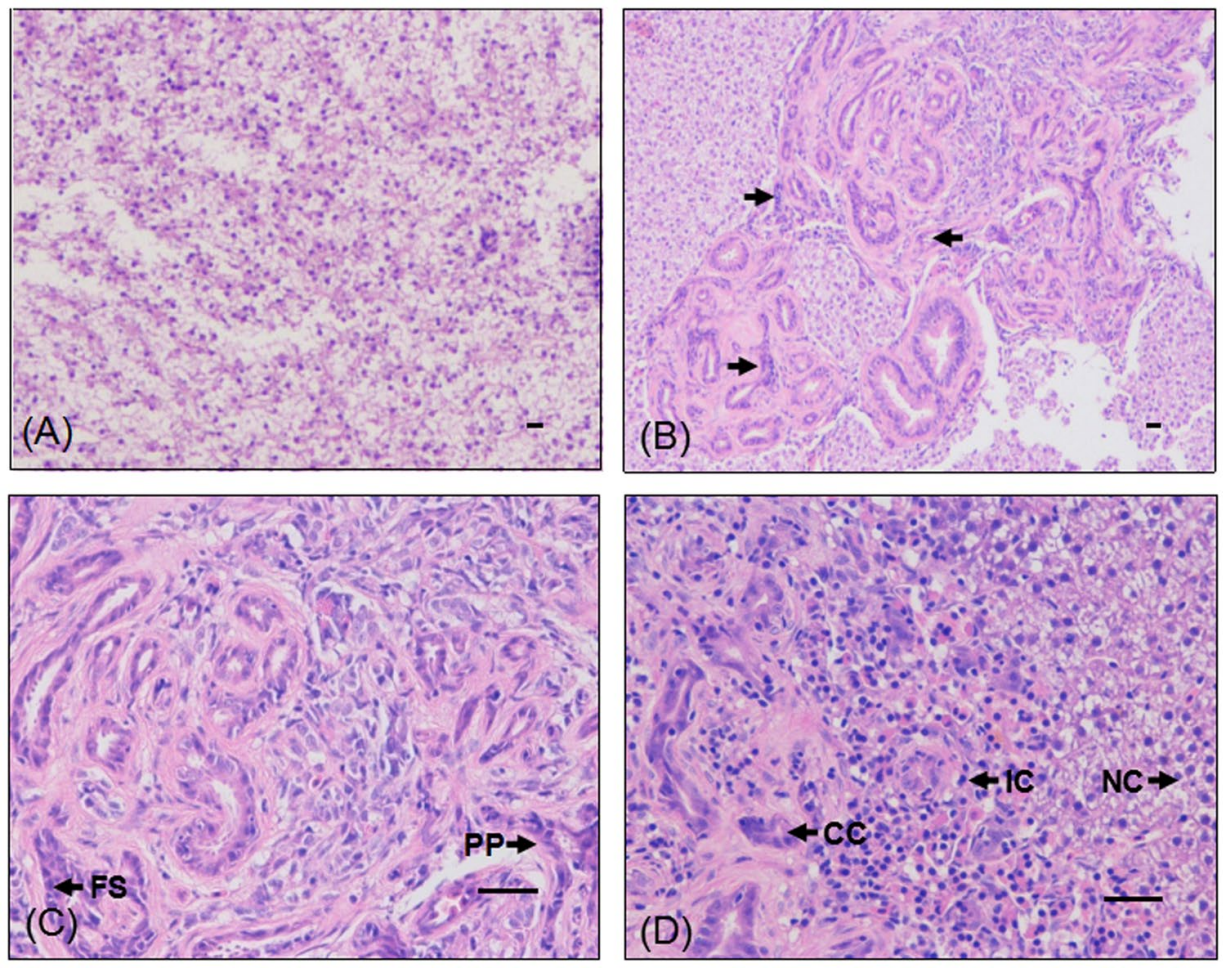
Histological and immunohistochemical examination of ICC in WT and *Tg*(*fabp10:nras* ^*61K*^) transgenic zebrafish (12 mpf) by hematoxylin and eosin (H&E) stain. Histological images of WT (**A**) and *Tg*(*fabp10:nras* ^*61K*^) transgenic zebrafish (**B**) at 12 mpf. (**C**) Histological analysis confirmed that the tumor was ICC. (**D**) Infiltration of cancer cell into the surrounding liver cell in the *Tg*(*fabp10:nras* ^*61K*^) transgenic zebrafish at 12 mpf. CC: cancer cell; FS: focally squamoid; IC: infiltrative type cell; NC: normal cell; PP: pleomorphic plump tumor cells; Scale bars: 50 µm.

**Figure 3:**
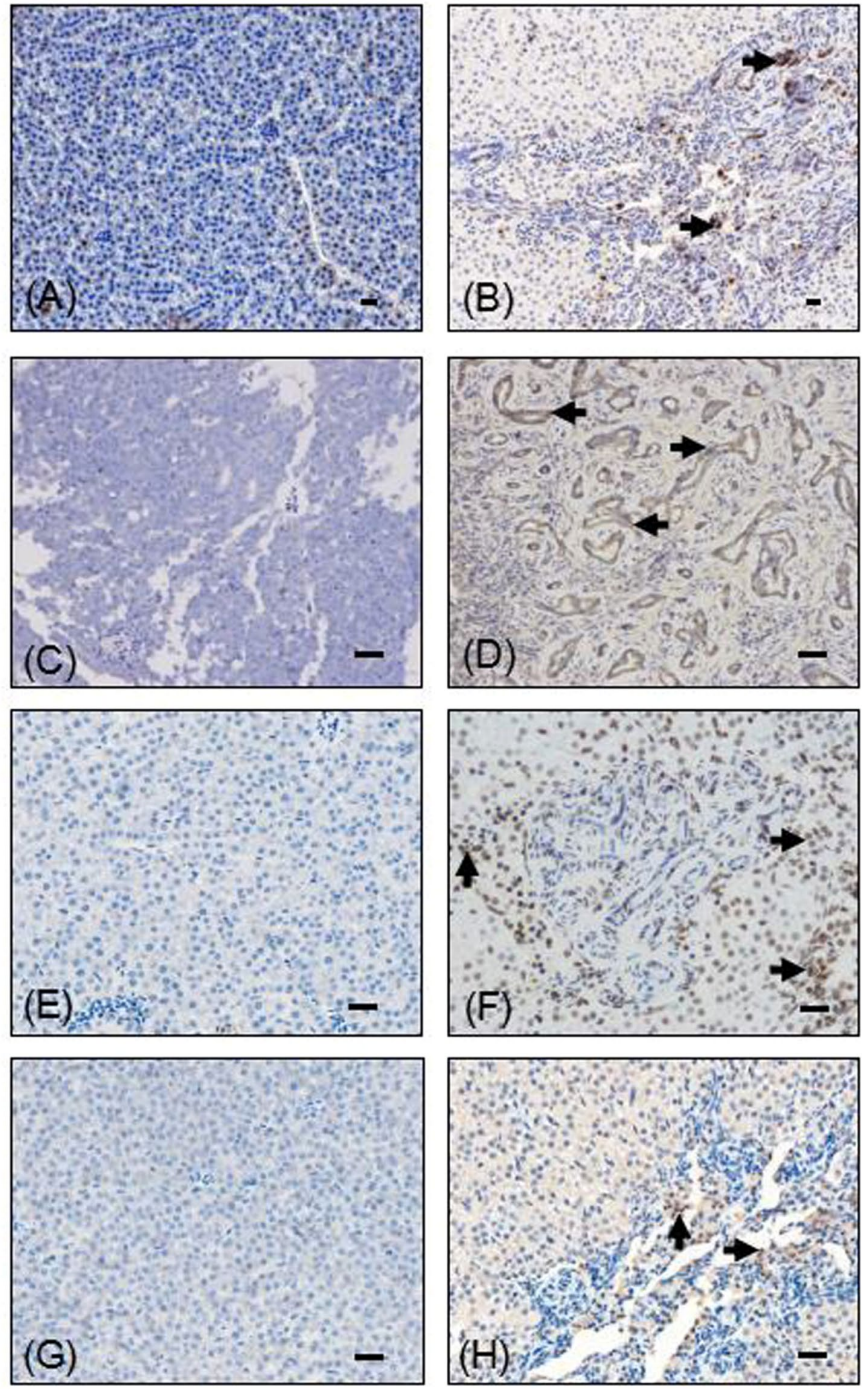
Immunohistochemical examination of ICC in WT and *Tg*(*fabp10:nras* ^*61K*^) transgenic zebrafish at 12 mpf. Immunohistochemical images of PCNA (**A**), CK19 (**C**), phosphorylated ERK (pERK) (**E**), phosphorylated MEK1/2 (pMEK1/2) (**G**) in WT and *Tg*(*fabp10:nras* ^*61K*^) transgenic zebrafish at 12 mpf. Tumor surrounding tissue of *Tg*(*fabp10:nras* ^*61K*^) transgenic zebrafish liver showing intense PCNA (**B**), CK19 (**D**), pERK (**F**), pMEK1/2 (**H**) immunostaining in *Tg*(*fabp10:nras* ^*61K*^) transgenic zebrafish at 12 mpf. Immunostaining signals in the bile duct of *Tg*(*fabp10:nras* ^*61K*^) transgenic zebrafish liver are indicated by arrowheads. Scale bars: 50 µm.

Gross morphological and histological analyses were performed in *Tg*(*fabp10:nras* ^*61K*^) transgenic zebrafish. Macroscopic liver nodules were detected in nine months old juveniles of the *Tg*(*fabp10:nras* ^*61K*^) transgenic zebrafish. The ratio of liver tumors was 67.2% (43/64), which presented histopathological features of intrahepatic cholangiocarcinoma (Fig. 1). The morbidity increased to 81.5% at 12 months of age (*n* = 65) and 86% at 24 months (*n* = 50) (Table 1). In addition, the homozygous F_4_ offspring (n = 128) showed 14%, 30%, 44% and 64% mortality by 9, 12, 15 and 18 months, respectively, compared with that of 10%, 15%, 19% and 24% in WT fish (n = 111) (Supplementary Fig. 1D). During tumor progression, hepatomegaly was observable in transgenic fishes. Obviously enlarged bellies (compared to the control) of *Tg*(*fabp10:nras* ^*61K*^) transgenic zebrafish were observed starting in 12 months old juveniles. Dissection of these fishes revealed that all had hepatomegaly (*n* = 43) and some even ascites (*n* = 3). Notably, histopathological diagnosis confirmed the ICC phenotypes, such as moderately pleomorphic plump tumor cells arranged in nests, trabeculae and ill-defined glands, focally squamoid appearing areas, and the infiltrative type pattern of growth into the surrounding liver (Fig. 2). Immunohistochemical (IHC) examination revealed that there was a sizable increase in PCNA (Fig. 3A,B) in ICC compared with WT.

**Table 1:** Percentages of tumors observed from *Tg*(*fabp10:nras* ^*61K*^) transgenic zebrafish at different time points.

Percentage of liver tumors in *nras* ^*61K*^ transgenic zebrafish				
3 mpf	6 mpf	9 mpf	12 mpf	24 mpf
0%(0/103)	0% (0/85)	67.2% (43/64)	81.5% (53/65)	86% (43/50)

Moreover, to validate ICC phenotype, ICC tumor marker called CK19, was monitored in *Tg*(*fabp10:nras* ^*61K*^) zebrafish livers. Strong positive signaling was observed using IHC (Fig. 3C,D). In addition, since Ras signals through MEK and ERK proteins via phosphorylation, we have identified the protein expression levels of phospho-MEK1/2 and phospho-ERK in WT and in *Tg*(*fabp10:nras* ^*61K*^) transgenic zebrafish livers. The IHC of liver sections showed apparently enhanced cytoplasmic and nuclear staining of phospho-MEK1/2 and phospho-ERK in ICC (Fig. 3E–H). Furthermore, *in vitro* study revealed that the green fluorescent protein (GFP) could not activate the phosphorylation of MEK and ERK (Supplementary Fig. 2).

### Transcriptomics of *Tg*(*fabp10:nras*^*61K*^) liver tumorigenesis

**Figure 4:**
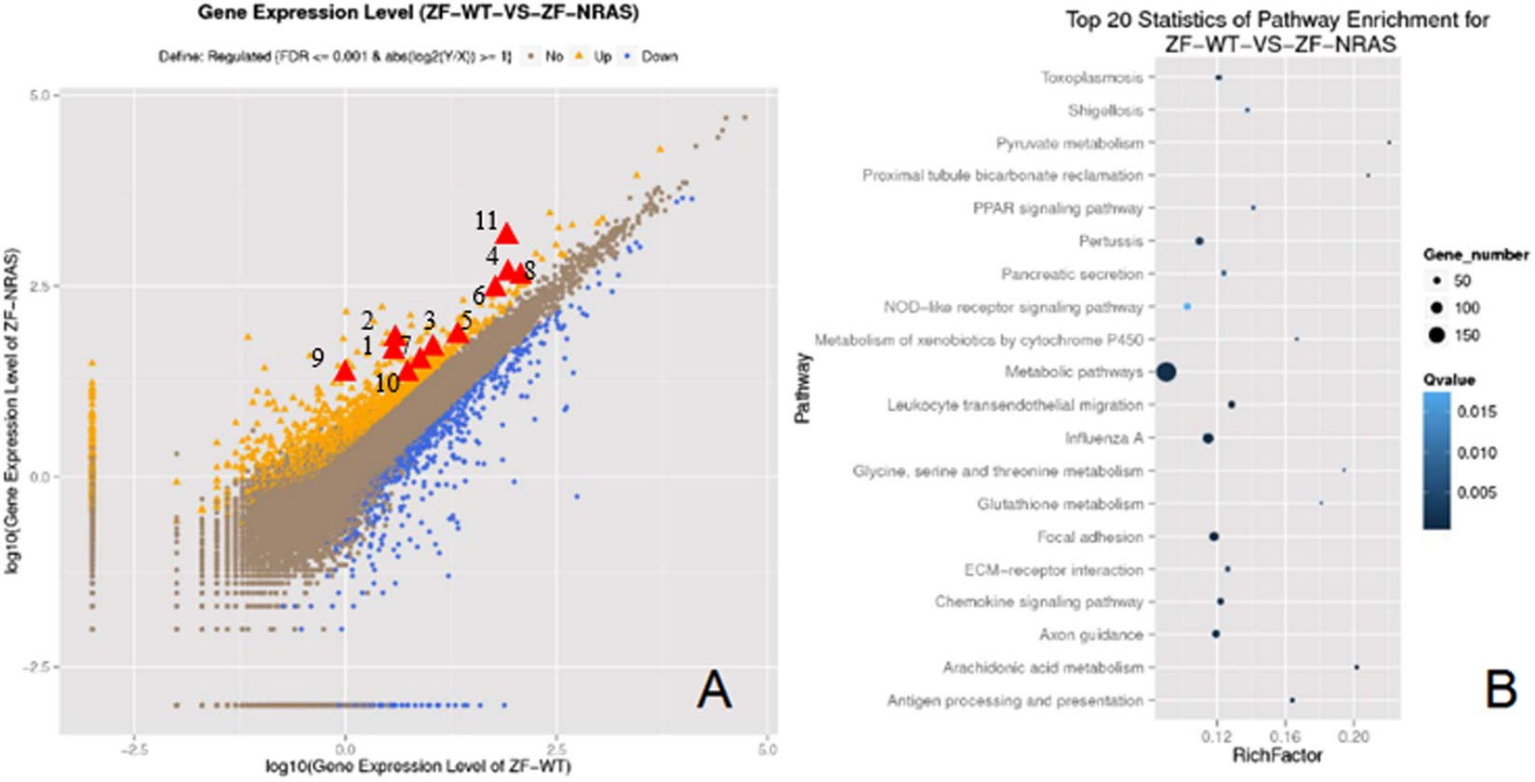
Transcriptomic analyses of *Tg*(*fabp10:nras* ^*61K*^) liver tumorigenesis. (**A**) Scatter plot comparing transcriptome profile of liver samples of WT vs. *Tg*(*fabp10:nras* ^*61K*^) transgenic zebrafish (12 mpf). Gene expression was measured by FPKM. The genes with the FDR ≤ 0.001 and log2(y/x) ≥ 1 are denoted as dots. The 1214 up-regulated genes are highlighted by yellow dots while 661 down-regulated genes are highlighted by bule dots. The 11 ICC marker genes are highlighted by red triangles. The number of representative genes, 1:*agr2*; 2: *ece2*; 3: *ehf*; 4: *epcam*; 5: *epha4*; 6: *eps8l3*; 7: *itgb6*; 8: *pdzk1ip1*; 9: *slc44a4*; 10: *slc6a*; 11: *spint2*. (**B**) Top 20 statistics of pathway enrichment for *Tg*(*fabp10:nras* ^*61K*^).

For the purpose of investigating the genes involved in *nras* ^*61K*^-induced ICC, we analysed the global gene expression in *Tg*(*fabp10:nras* ^*61K*^) transgenic zebrafish livers. By applying the data filtering criteria (the adaptor sequences; unknown bases more than 10%; the percentage of no more than Q 5 bases is over 50% in a read) have been filtered, we obtained 48.6 million 90-bp sequence reads per sample. Nevertheless, approximately 34.6 in WT and 34.9 million reads in *Tg*(*fabp10:nras* ^*61K*^) transgenic zebrafish belonged to the zebrafish genome by analysis as described by *Bowtie* ^24^ along with enrichment analysis with Noiseq.^25^. 661 down-regulated- and 1214 up-regulated- differentially expressed genes in *Tg*(*fabp10:nras* ^*61K*^) transgenic zebrafish were observed (Fig. 4A and Supplementary Table 1). Functions of different gene expressions in the ICC were involved in metabolic, focal adhesion, and chemokine signaling pathways (Fig. 4B).

Moreover, Gene Set Enrichment Analysis (GSEA) was performed to analyze the similarities among the gene-expression profiles from the *Tg*(*fabp10:nras* ^*61K*^) transgenic zebrafish and two previous studies reported by the Obama^26^ and Jinawath^27^ groups. Both studies determined gene-expression profiles in tumor samples from patients with intrahepatic cholangiocarcinoma (n = 25 for Obama group; n = 40 for Jinawath group). Our results revealed that three upregulated genes and 24 downregulated genes derived from the *Tg*(*fabp10:nras* ^*61K*^) transgenic zebrafish model showed similarity with the significantly enriched gene sets in these two studies (Supplementary Table 2). In addition, previous studies showed that 25 significantly mutated genes^28^ and top 42 up-regulated genes^29^ in ICC patients are likely to be good candidates for ICC markers. In this study, eleven similar up-regulated markers were identified (log_2_ fold change ≥1 and P ≤ 0.05) and validated using the RT-qPCR (Table 2 and Supplementary Fig. 3), suggesting that the liver morphological changes are in connection with the alterations of gene expression.

**Table 2:** Eleven potential ICC marker genes were obviously up-regulated in *nras* ^*61K*^ transgenic zebrafish.

EntrezGen IDs from Zebrafish	Gene Symbol	Gene Name	Expression Changes in Zebrafish ICC (log_2_ ratio)	Expression p-value	qRT-PCR (log_2_ ratio)	RT p-value
ENSDARG00000070480	*agr2* ^*#*^	Anterior gradient 2	3.89	3.28E-20	4.2	<0.0001
ENSDARG00000087841	*ece2* ^*^	Endothelin converting enzyme 2	3.36	1.21E-13	4.23	<0.0001
ENSDARG00000052115	*ehf* ^*#*^	Ets homologous factor	2.16	1.30E-08	1.8	<0.0001
ENSDARG00000040534	*epcam* ^*#*^	Epithelial cell adhesion molecule	2.51	3.63E-58	2.08	0.0001
ENSDARG00000011600	*epha4* ^*^	Eph receptor A4	3.62	2.44E-25	5.07	<0.0001
ENSDARG00000077296	*eps8l3* ^*#*^	EPS8-like 3	2.18	1.40E-25	3.4	<0.0001
ENSDARG00000002494	*itgb6* ^*#*^	Integrin, beta 6	2.78	1.41E-08	2.47	<0.0001
ENSDARG00000017127	*pdzk1ip1* ^*#*^	PDZK1 interacting protein 1	1.75	9.51E-34	1.12	<0.0001
ENSDARG00000102381	*slc44a4* ^*#*^	Solute carrier family 44, member 4	4.75	8.07E-08	3.26	<0.0001
ENSDARG00000029866	*slc6a* ^*#*^	Solute carrier family 6 (amino acid transporter), member 14	3.65	4.81E-42	2.85	<0.0001
ENSDARG00000069476	*spint2* ^*#*^	Serine peptidase inhibitor, Kunitz type, 2	1.98	4.05E-91	1.18	0.0004

^*^Those genes are highly mutated in human ICC.

^#^Those genes are highly expressed in human ICC.

### Landscape of the methylome analysis in liver samples of *Tg*(*fabp10:nras*^*61K*^) zebrafish

**Figure 5:**
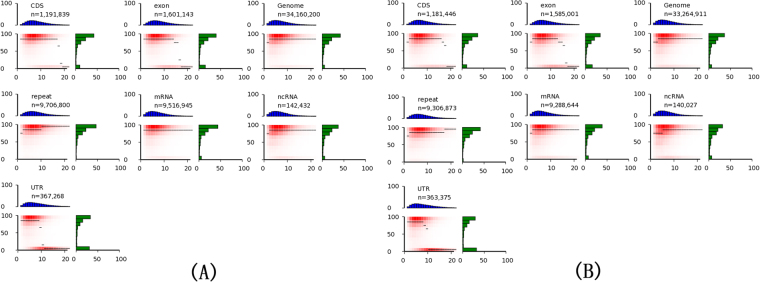
Landscape of methylome in WT and *Tg*(*fabp10:nras* ^*61K*^) transgenic zebrafish (12 mpf). Heat maps show distinct methylation and CG density patterns for different genomic features in WT (**A**) and *Tg*(*fabp10:nras* ^*61K*^) transgenic zebrafish (**B**). Each panel represents a separate feature, and *n* refers to the number of analyzed CGs (per-strand depth ≥10) within that feature. CG density (*x*-axis) is defined as the number of CG dinucleotides in 200 bp windows. Methylation level (*y*-axis) is defined as the mean methylation level of cytosines in CGs. The thin black lines within each heat map denote the median methylation level of CGs at the given local density. The red gradient hypo indicates the abundance of CGs that fall into bins of given methylation levels and CG densities. The blue bar charts above each heat map show the distribution of CG densities, projected onto the *x*-axis of the heat maps. The green bar charts to the right of the heat maps show the distribution of methylation levels, projected onto the *y*-axis of the heat maps.

In order to investigate the abnormal DNA methylation in the transgenic ICC zebrafish, global analysis of the liver samples of *Tg*(*fabp10:nras* ^*61K*^) transgenic and WT zebrafish methylomes were performed. For the transgenic samples, totally 760.5 M raw reads were produced. After removing low-quality and clonal reads, we obtained 608.3 M effective reads, and the sequence yield for final analysis was 54.3 gigabase pairs (Gb), covering 90.75% of all cytosines in the genome with an average depth of 22.77 per strand. Initially we observed overall genome-wide methylation levels of 72.49% at CG, 0.54% at CHG and 0.51% at CHH sites (H = A, C or T), indicating higher CG methylation than non-CG methylation. The overall CG methylation level of transgenic zebrafish was lower than that in WT zebrafish (75.82%), but it was still considered to be relatively high. Methylation distribution displayed the similar bimodal (6.37% hypo (<20%) methylated, 62.2% hyper (>80%) methylated) methylation pattern to the WT zebrafish liver cells (5.47% and 62.5%, respectively). This indicates that there was no bias introduced by the whole-genome approach used here. The heat map below presents the distribution of methylation levels in each genomic feature of the liver samples of *Tg*(*fabp10:nras* ^*61K*^) transgenic zebrafish (Fig. 5). As shown in Fig. 5., the thin black lines within each heat map denote the median methylation level of CGs at the given local density. Our result show different median methylation level of CGs in the non-coding RNA sequences, exons and repeat elements of *Tg*(*fabp10:nras* ^*61K*^) transgenic zebrafish compared with WT, suggesting it may have a role in the tumorigenesis. Furthermore, all transcriptional units were divided into six distinct functional elements to demonstrate the changes of methylation level. Our result indicate that two discrete switch over zones, upstream of the TSS and the exon 1, demarcate the transition from hypo- to hypermethylation in the inverse relationship between promoter and gene-body methylation and expression both in liver samples of *Tg*(*fabp10:nras* ^*61K*^) transgenic and WT zebrafish (data not shown).

**Figure 6:**
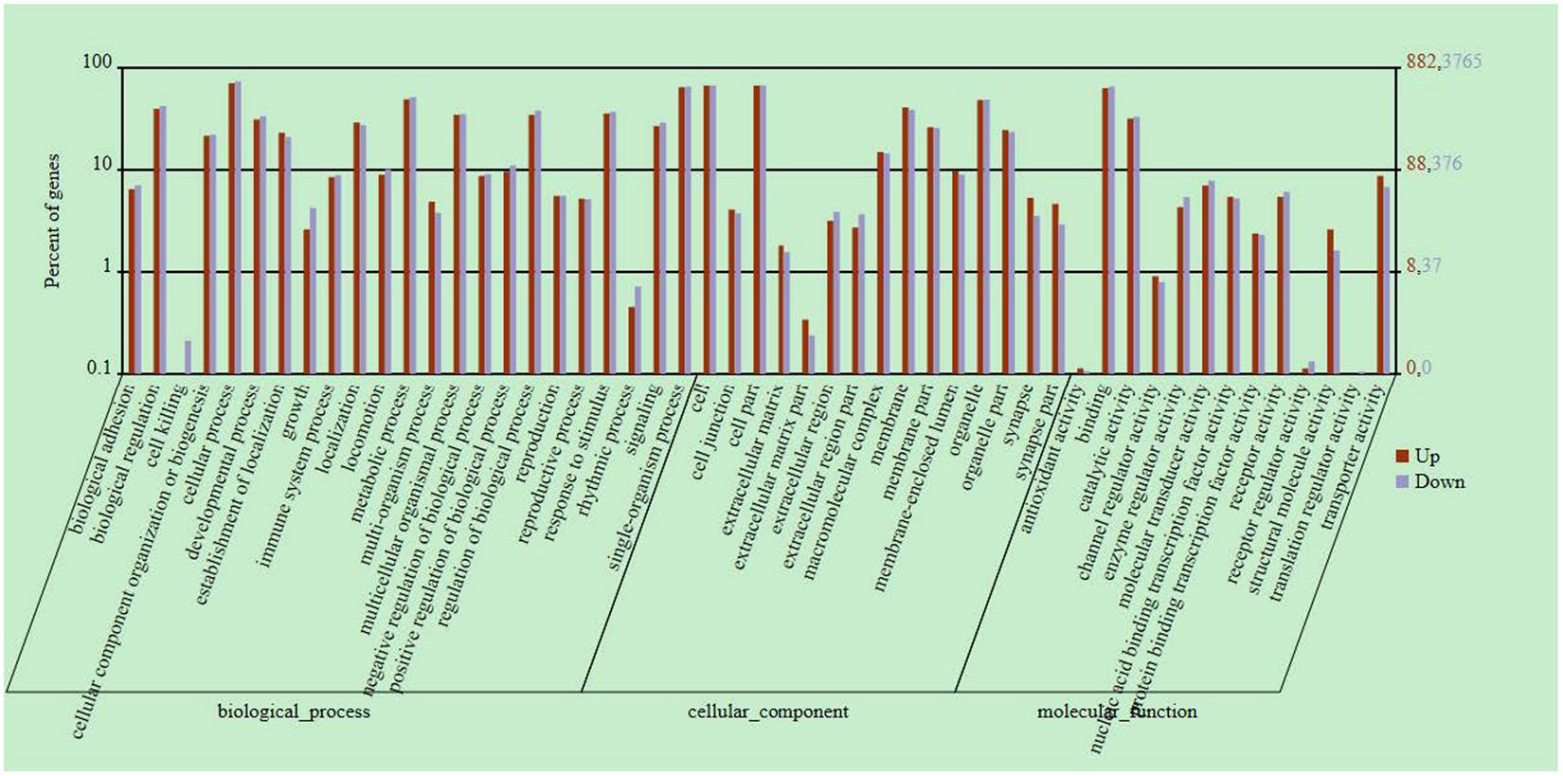
Annotation analysis of methylated genes in *Tg*(*fabp10:nras* ^*61K*^) transgenic zebrafish (12 mpf). Annotation of methylated genes with WEGO. Of all the genes that have GO annotations, 8,599 methylated genes show significant enrichment difference (P < 0.05, χ2 test) compared with total analyzed genes. Annotations are grouped by molecular function or biological process based on the gene ontology database (http://www.geneontology.org/). Gene numbers and percentages are listed for each category.

To functionally categorize the methylated genes of *Tg*(*fabp10:nras* ^*61K*^) transgenic and normal samples, we performed the BGI WEGO (Web Gene Ontology Annotation Plotting) analysis, which revealed significant differences (Fig. 6). Compared with normal samples, methylated genes in *Tg*(*fabp10:nras* ^*61K*^) transgenic zebrafish were enriched in cellular component and binding activities. As for biological processes and cellular component, they tend to be enriched in functions associated with cellular process, single-organism process and metabolic process (Fig. 6).

### Comparison of methylome and transcriptomic profiles

To further investigate the contribution of epigenetic alterations to the pathogenesis of ICC. We compared the methylome and transcriptomic profiles with the finding that when the CG methylation up-regulated, there were 72 genes up-regulated and 44 genes down-regulated at transcription level, while 924 genes remained unchanged. On the contrary, when the CG methylation was down-regulated, the number of up-regulated, down-regulated and unchanged genes at transcription level were 406, 182 and 4011, respectively (Supplementary Fig. 4). Particularly, the methylation level of 11 obviously up-regulated potential ICC marker genes mentioned above was investigated and the result revealed that three genes were changed, including *ehf*, *epha4* and *itgb6* (Table 3). Both *epha4* and *itgb6* have one specific CpG site, while *ehf* has two specific CpG sites. Moreover, we used bisulfite sequencing PCR to assess the methylation status of the CpG islands in these genes, and found that these CpG islands were hypomethylated (Supplementary Table 3).

**Table 3:** Three potential ICC marker genes were hypomethylated in *nras* ^*61K*^ transgenic zebrafish.

EntrezGen IDs from Zebrafish	Gene Symbol	Gene Name	CG down
ENSDARG00000070480	*agr2*	Anterior gradient 2	
ENSDARG00000087841	*ece2*	Endothelin converting enzyme 2	
ENSDARG00000052115	*ehf*	Ets homologous factor	+
ENSDARG00000040534	*epcam*	Epithelial cell adhesion molecule	
ENSDARG00000011600	*epha4*	Eph receptor A4	+
ENSDARG00000077296	*eps8l3*	EPS8-like 3	
ENSDARG00000002494	*itgb6*	Integrin, beta 6	+
ENSDARG00000017127	*pdzk1ip1*	PDZK1 interacting protein 1	
ENSDARG00000102381	*slc44a4*	Solute carrier family 44, member 4	
ENSDARG00000029866	*slc6a*	Solute carrier family 6 (amino acid transporter), member 14	
ENSDARG00000069476	*spint2*	Serine peptidase inhibitor, Kunitz type, 2	

## Discussion

Intrahepatic cholangiocarcinoma is a highly malignant liver cancer that affects 5–10% of all primary liver cancers. ICC arises in the epithelial bile ducts in the biliary tree and shows quite different characteristics from HCC. Although it is a relatively rare malignancy, it deserves immediate attention because of its steady and substantial increase ranging from 0.32 to 0.85 per 100,000 people worldwide over the last 30 years^30,31^. It is particularly prevalent in Asian countries, for instance, the prevalence of ICC in Thailand is 96 per 100,000 people, which is 100-fold higher than the worldwide average^32^. Unfortunately, ICC is very difficult to diagnose and is usually associated with high mortality due to its late clinical presentation and the lack of effective non-surgical therapeutics^33^.

Here we report an *in vivo* ICC model using the transgenic overexpression of oncogene *nras* ^*61K*^ in zebrafish. This work is reminiscent of a previous study that reported a HCC transgenic model which constitutively expresses the *kras* ^*V12*^ oncogene in the zebrafish liver^6^. Intriguingly, our study uncovered that even using the same liver-specific *fabp10* promoter, a different kind of *ras* oncogene would lead to distinct type of liver cancer. One explanation for the phenotypic differences between *kras* ^*V12*^ and *nras* ^*61K*^ is that they are expressed at different levels within specific cell types^34^. Another previous study reported that hepatocytes may be capable of changing their fate to biliary lineage cells regardless of their position in the hepatic lobule^35^. It is suggested that *nras* ^*61K*^ may contribute to this process^35^. However, further studies are necessary to determine whether *nras* ^*61K*^ is responsible for the conversion of hepatocytes to the biliary lineage cells, and next, to induce the malignant transformation to the intrahepatic cholangiocarcinoma.

Transcriptomic analyses show that genes related to metabolic pathways are most abundant in the ICC samples, identifying the importance of metabolic modulation during the tumorigenesis in ICC. It was reported that *tmprss4* (Transmembrane protease serine 4) is up-regulated in a broad spectrum of cancers^36^. Overexpression of *tmprss4* significantly promotes the invasion, migration, adhesion and metastasis in HCC^37^. In our study, it was demonstrated that *tmprss4* might serve as an evolutionarily conserved marker in liver cancer. Furthermore, several gene signatures which acted as potential markers for ICC^29,30^ were analysed in this study. Moreover, our data uncovered that 11 of these potential ICC marker genes (Table 3) were obviously up-regulated in the *Tg*(*fabp10:nras* ^*61K*^) transgenic zebrafish, indicating that these genes may play critical roles in the *nras* ^*61K*^ liver tumorigenesis and that our model provides a good platform for ICC studies.

Deregulation of developmental genes by hypomethylation of CG islands appears to be one of the major factors driving tumorigenesis^23^. Recently, several studies reported DNA methylation in *nras*-mutated cancers. For instance, Furlan *et al*.^38^ found that DNA demethylation together with specific *nras* mutations drives the early steps of oxidative damage colorectal tumourigenesis. Another study reported that the hypomethylated genes may play important roles in the pathogenesis of *nras* ^*61K*^-mutated melanoma^21^. Therefore, we explored the genome-wide methylation of *Tg*(*fabp10:nras* ^*61K*^) transgenic zebrafish. As shown in Fig. 6, methylated genes in *Tg*(*fabp10:nras* ^*61K*^) transgenic fish were observed, associated with cellular components, binding activities and biological processes such as cellular process, metabolic process and single-organism process. It is of note that genes involved in metabolic process are the largest group in differentially expressed genes, which suggests that genes in metabolic pathways are of considerable significance to the formation of ICC through methylation. Furthermore, our result uncovered a rich landscape of distinct epigenomic features such as repeat elements, coding, and non-coding sequences, in the livers of *Tg*(*fabp10:nras* ^*61K*^) transgenic zebrafish. For example, exons were clearly distinguishable from introns by elevated methylation levels, delimited by sharp intron-exon boundaries. This finding was consistent with some recent reports that exons can be determined by epigenetic marks^39,40^. Our data provided biological information to analyse features that have previously been difficult to assess such as repeat elements^41^.

In addition, it is very crucial in terms of cancer diagnosis that cancer-/cancer-stage-specific biomarkers are identified^42^. In this study, one of our goals is to identify ICC-related biomarkers by virtue of methylome and transcriptomic analysis. Our results showed that three potential ICC marker genes were hypomethylated in liver samples of the 12 months post fertilization (mpf) *Tg*(*fabp10:nras* ^*61K*^) transgenic fish. One notable example is *epha4* (EPH tyrosine kinase receptor A4), a member of the receptor tyrosine kinases gene family, modulating the epithelial-mesenchymal transition process, which was hypomethylated in our results. Previous study reported that *epha4* promotes cell proliferation and migration in glioblastoma^43^. However, *epha4* mRNA is significantly down-regulated in HCC tissues with the ability to inhibit the cancer cell migration and invasion, and promoting cell adhesion^44^. Interestingly, *epha4* was up-regulated (5.07-log_2_ fold change, *p* value < 0.0001) in our ICC samples, suggesting the exsistence of different mechanisms in ICC, prompting further investigations.

Moreover, hypomethylated loci featured prominently in gene bodies (mainly in introns) but not promoter regions, which is similar to the previous report of the *nras* ^*61K*^-mutated melanoma^21^. One explanation is that the CpG-rich sequences in these intron of the oncogenes were potential mediators linking the oncogene and its transcription factor. For instance, Lee *et al*.^45^ reported that the transcription factor MZF1, which binds to CpGs in the first intron of *PRAME* (preferentially expressed antigen in melanoma) gene, could induce the up-regulation of PRAME by increasing DNA hypomethylation, and then promote the proliferation of melanoma cells. However, the mechanisms of the DNA hypomethylation in these ICC marker genes remain to be elucidated in the future.

In summary, we successfully constructed a *Tg*(*fabp10:nras* ^*61K*^) transgenic model and created an ICC animal model. Our model provides a good tool to investigate the molecular events involved in the progression of bile duct neoplasms. The methodologies utilized to study the whole-gene methylation and transcriptomes during the development of ICC supplied the tools to study the meachnisms of ICC. Consequently, the final purpose of research in this field is to find and screen therapeutic drugs, and we believe that the ICC model can be applied to high-throughput screen the anti-ICC drugs.

## Materials and Methods

### Vector construction and production of *Tg*(*fabp10:nras*^*61K*^) transgenic zebrafish lines

The liver-driver construction was made by the insertion of zebrafish liver-specific fatty acid-binding protein (*fabp10*) promotor at the *Apal* I and *Nhe* I sites into the vector pAcGFP1-N1 (Clontech, Mountain View, CA, 632469). Human *nras* ^*61K*^ gene was inserted into the vector pAcGFP1-N1 at the *Sal* I and *BamH* I sites. The cDNA encoding human *nras* ^*61K*^ gene was inserted at the C-terminal of the liver-specific *fabp10* promotor and the N-terminal of the green fluorescent protein (GFP) provided by the pAcGFP1-N1 vector. Linearized plasmid of pAcGFP1-N1-*fabp10-nras* ^*61K*^ was injected into one-cell embryos of the AB strain zebrafish. Experiments involving zebrafish in this study were approved by the Animal Research and Ethics Committees of Institute of Hydrobiology, Chinese Academy of Sciences, and all experiments were conducted in accordance with the guidelines of the committees.

### Tumor screening and histopathological analysis

The GFP-positive embryos were raised to adults and out-crossed to wild-type fish for testing germline transmission. Transgenic fish were dissected to expose the abdominal area and liver morphology observed under the SMZ1600 stereomicroscope (Nikon, Tokyo, Japan). Liver tumor in transgenic fish was defined as GFP-marked liver which was enlarged to at least twice the size of a WT normal liver. Liver samples were then fixed in 4% paraformaldehyde (PFA) for at least 24 hours by dehydration through a series of graded ethanol solutions and embedding in paraffin. Four µm- thick sections were cut and stained with hematoxylin and eosin (H&E) for histological analysis. The following antibodies were used for immunohistochemical study: anti-CK19 (Abcam, Cambridge, UK, ab9221), anti-PCNA (Servicebio, Wuhan, China, GB13010–1), anti-MEK1 (HuaAn, Hangzhou, China, ET1603-20), anti-MEK2 (HuaAn, Hangzhou, China, ET1612-6), anti-ERK (Servicebio, Wuhan, China, GB13003-1), anti-pMEK1/2 (Cell Signaling, Danvers, MA, #9154), anti-pERK (Servicebio, Wuhan, China, GB13004-1).

### Western blot analysis

The 293 T cells were collected after trypsinization and resuspended in mammosphere medium. The cells were then transfected with 100 nmol/liter pAc-GFP-*nras* ^*61K*^ or pAc-GFP as control. The liver tissues from the 3, 6, 9 and 12 mpf *Tg*(*fabp10:nras* ^*61K*^) transgenic zebrafish and WT (12 mpf) were collected respectively, and the livers of four fish were pooled to generate one sample. The samples were lysed with RIPA lysis buffer [20 mM Tris/HCl (pH 7.4), 150 mM NaCl, 0.5% Nonidet P40 and 1 × protease inhibitor cocktail (Roche, Penzber, Germany)], and the lysates were incubated with the suitable antibody including anti-β actin (AB clonal, Wuhan, China, AE012), anti-EGFP (Santa Cruz, CA, SC-47778), anti-MEK1, anti-MEK2, anti-ERK, anti-pMEK1/2, anti-pERK.

### Identification and mapping of integration sites

Genomic DNA was isolated from the F_3_ transgenic line and digested with *Dra I*, *EcoR V*, *Stu I*, and *Pvu II*. The digested DNA was purified and ligated with GenomeWalker^TM^ adaptor (Clontech, Mountain View, CA, 638904). Primary PCR was performed using primer AP1 and gene-specific primer (GSP1: 5′-AAA TAC TCA GAG CAG CCC ATC TGG-3′). The PCR product was 50- fold diluted as a template and secondary PCR was performed using AP2 and gene-specific primer (GSP2: 5′-GTC GAC TCC CTT TAG TGA GGG TTA-3′). PCR products larger than 1 kb were cloned and sequenced. These sequence reads were mapped to the zebrafish genome using BLASTN (GRCz10, November 2015 freeze in the Ensembl).

### Transcriptome sequencing and analysis

Liver tissues from both WT and *Tg*(*fabp10:nras* ^*61K*^) transgenic zebrafish were collected at 12 mpf. Livers of four fish were pooled to generate one sample. Total RNA was purified by beads containing oligo (dT). Purified mRNA was then fragmented in fragmentation buffer. Using these short fragments as templates, random hexamer-primers were applied to synthesize the first-strand cDNA. Briefly, second-strand cDNA was synthesized using buffer, dNTPs, RNase H and DNA polymerase I. Short double-stranded cDNA fragments were purified with a QIAquick PCR extraction kit (Qiagen, Valencia, CA, 28104) and eluted with EB buffer for end repair and the addition of an ‘A’ base. Next, short fragments were ligated to Illumina sequencing adaptors. DNA fragments of a selected size were gel-purified and amplified by PCR. The amplified library was sequenced on an Illumina HiSeq™ 2000 sequencing machine (Qiagen, Valencia, CA). The details of the experiment were as follows: Expected library size: 200 bp; Read length: 90 nt; and Sequencing strategy: paired-end sequencing. The primary analysis was performed with the Illumina HiSeq^TM^ 2000 (Illumina, San Diego, CA). The GSEA analysis was performed as described previously^46^.

### Quantitative reverse-transcription polymerase chain reaction

Total RNA from liver tissue of each individual fish was isolated by means of a SV total RNA isolation system kit (Promega, Madison, WI, Z3100). Then, 4 µg of isolated total RNA was transcribed to cDNA through the RevertAid^TM^ First Strand cDNA Synthesis Kit (Fermentas, Waltham, MA K1662) with oligo-dT primer. Quantitative RT-PCR was performed using the Fast start universal SYBR Green Master Mix (Roche, Penzber, Germany, 11750800) with an Applied Biosystems StepOne Real-Time PCR System (AB, Foster City, CA). Primers for PCR were designed via an online software IDT Real Time PCR Tools (http://www.idtdna.com/scitools/Applications/RealTimePCR/). Primer sequences are listed in Supplementary Table 4.

### DNA isolation, BS-seq library construction and sequencing

Genomic DNA was isolated from liver tissues of both WT and *Tg*(*fabp10:nras* ^*61K*^) transgenic zebrafish (12 mpf). Livers of four fish were pooled to generate one sample. Then 5 µg DNA was used to do the bisulfite conversion and bisulfite sequencing (BS-seq) using a modified NH_4_HSO_3_-based protocol^47^. Paired-end library construction and sequencing were implemented using Illumina HiSeq. 2000, according to the manufacturer’s instructions (Illumina, San Diego, CA). We also mixed 25 ng cl857 Sam7 Lambda DNA in each sample to utilize as conversion quality control for each library.

### BS-seq analysis

BS-seq reads were mapped to the reference genome using SOAP2^48^ as described^49^, allowing up to 6 mismatches for 90 bp paired-end reads. Multiple reads mapping to the same position were regarded as PCR duplicates, and only one of them was kept. However, bases with a quality score less than 20 were not considered for subsequent analysis.

The error rate of each library (sum of the non conversion rate and T/C sequencing errors) was calculated as the total number of sequenced Cs divided by the total sequencing depth for sites corresponding to Cs in the Lambda genome. The error rate for each library was approximately 0.5%. To distinguish true mCs from false positives, we applied a model based on the binomial distribution B (n, p) following^49^, and only the mCs with FDR^50^ adjusting to P-values less than 0.01 were considered true positives. All experiments were performed in triplicate.

### Estimation of methylation level and bisulfite DNA sequencing PCR analysis

To estimate the methylation level of a single base accurately, the CpG cytosines with a per-strand depth of less than 4 have been excluded from the analysis. Methylation level of an individual CpG was determined by the number of reads containing a C at the site of interest divided by the total number of reads covering the site. Meanwhile, methylation level of a specific region was determined by the sum of methylation levels of individual CpGs in the region divided by the total number of overed CpGs in this region.

Genomic DNA was isolated from liver tissues of both WT and *Tg*(*fabp10:nras* ^*61K*^) transgenic zebrafish (12 mpf). Livers of four fish were pooled to generate one sample. The DNA samples were treated with sodium bisulfite to convert cytosine to uracil using the BisulFlash™ DNA Modification kit (Epigentek, USA, P-1026-050) according to the manufacturer’s instruction. Bisulfite sequencing was performed as described previously^51^, and the amplified bisulfite sequencing PCR products were sequenced to determine the methylation status of each CpG site. Primer sequences are shown in Supplementary Table 5.

### Statistical analysis

Differentially expressed genes (DEGs) were selected by applying the statistical significance threshold of log2-fold >1.0 or <−1.0, with the false discovery rate (FDR) threshold set at <0.01 and p < 0.01. Gene Ontology (GO) analysis was applied to analyze the primary functions of the DEGs using MetaCore software (GeneGo). The data are reported as average results of three independent experiments.

RT-PCR data are reported as mean + S.E.M. of three independent experiments. Statistical analysis (unpaired t-test) was performed using GraphPad Prism 5 (GraphPad Software Inc.).

## Electronic supplementary material

Supplementary Table 1.

Supplementary Table 2.

Supplementary Information
