# Supplementary material for: The construction of intrahepatic cholangiocarcinoma model in zebrafish
Source: Sci Rep. 2017 Oct 17;7:13419. doi: 10.1038/s41598-017-13815-0 (PMC5645375; doi:10.1038/s41598-017-13815-0)
Supplement: Supplementary file 3 — Supplementary Information [file 41598_2017_13815_MOESM3_ESM.pdf]

Supplementary Information

The construction of intrahepatic cholangiocarcinoma model in zebrafish

Jing Wang[1], Xiaoqian Leng[1,2], Guiping Wang[1], Xiaoyang Wan[1] and Hong Cao[1,*]

[1]State Key Laboratory of Freshwater Ecology and Biotechnology, Institute of Hydrobiology, Chinese Academy of Sciences, Wuhan 430072, China

[2]Key Laboratory of Freshwater Biodiversity Conservation, Ministry of Agriculture of China, Yangtze River Fisheries Research Institute, Chinese Academy of Fisheries Science, Wuhan 430223, China

*Author for correspondence (regancao@ihb.ac.cn)

Supplementary Figure 1

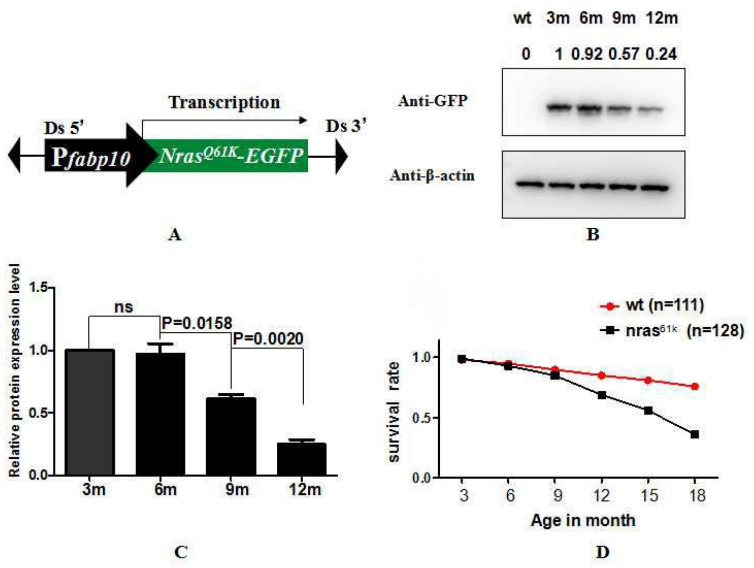

Fig. 1. Generation of *Tg*(*fabp10:nras*^{61K}) transgenic zebrafish.

(A) Schematic diagram of the *Tg*(*fabp10:nras*^{61K}) transgenic zebrafish. The liver-specific *nras*^{61K}*-EGFP* gene was inserted into the C-terminus of the liver-driver promoter pfabp10. (B) Western blots showed that GFP-NRAS^{61K} were expressed in the live samples of *Tg*(*fabp10:nras*^{61K}) transgenic zebrafish at 3, 6, 9 and 12 mpf compared with WT (12 mpf). (C) The relative protein expression level of GFP-NRAS^{61K} in the live samples of *Tg*(*fabp10:nras*^{61K}) transgenic zebrafish at 3, 6, 9 and 12 mpf. (D) The survival curves of the homozygous *Tg*(*fabp10:nras*^{61K}) transgenic zebrafish (n=128) and WT siblings as control (n=111).

Supplementary Figure 2

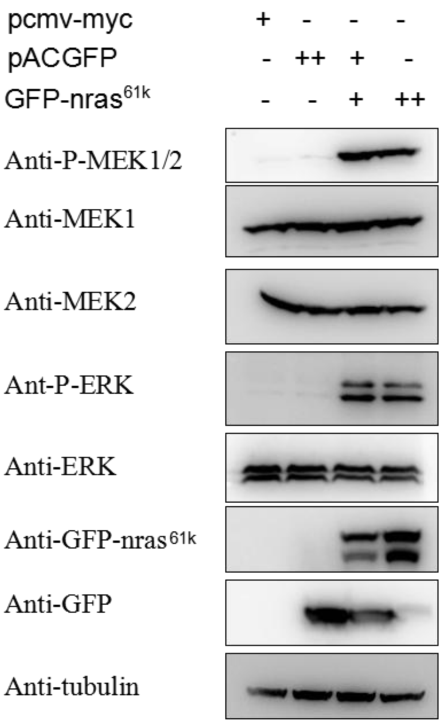

Fig. 2. pMEK1/2 and pERK were induced by GFP-NRAS^{61K} in 293T cells.

Western blots showed that pMEK1/2 and pERK were induced after transfection of pAc-GFP-*nras*^{61K} in 293T cells compared with controls.

Supplementary Figure 3

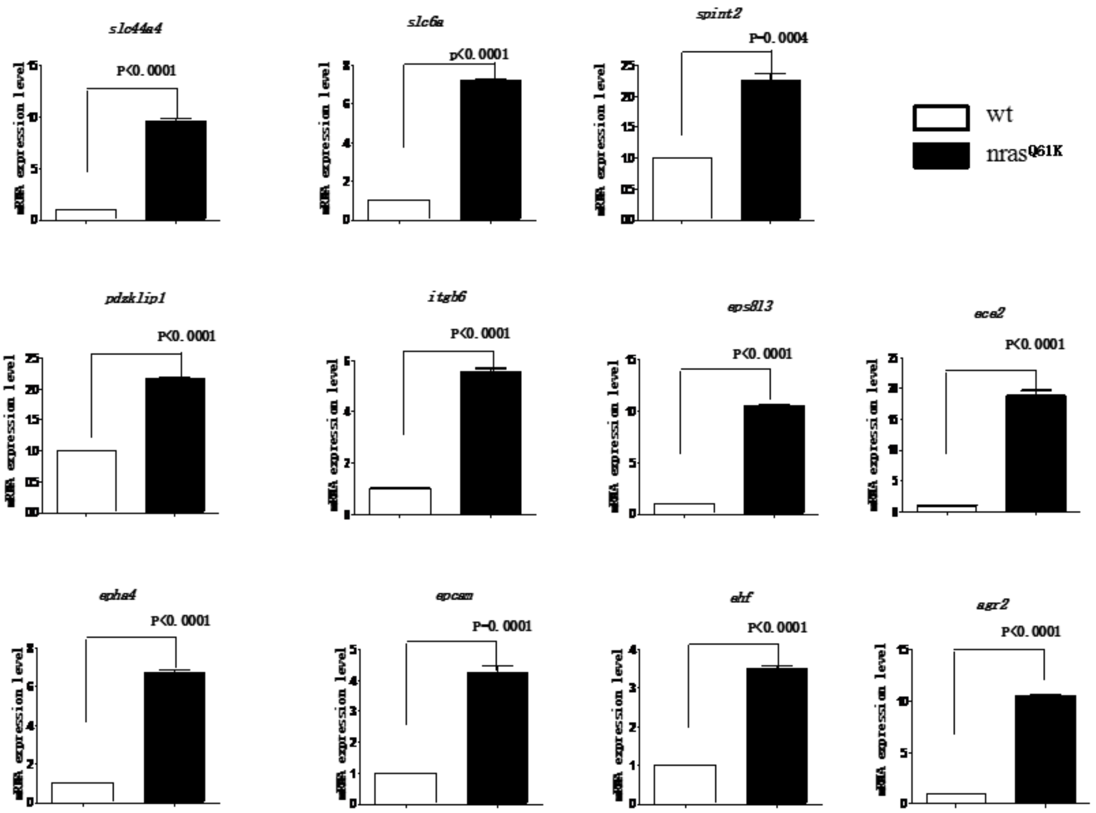

Fig. 3. Determination of expression levels of 11 ICC markers by qRT-PCR in the liver of WT and *Tg*(*fabp10:nras*^{61K}) transgenic zebrafish (12 mpf).

Determination of expression levels of 11 ICC markers (log2 fold change≥1 and P≤0.05) by qRT-PCR in the liver of WT and *Tg*(*fabp10:nras*^{61K}) transgenic zebrafish (12 mpf). The expression levels of these genes in each WT and transgenic liver sample were first measured and normalized with the expression level of β-actin (n=4 each). The log2 fold changes in expression in the transgenic samples as compared with matched WT sample are presented.

Supplementary Figure 4

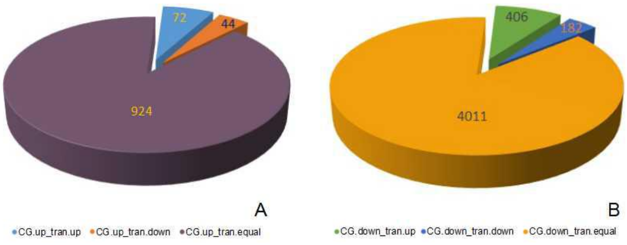

Fig. 4. Comparative analysis of the methylome and transcriptomic profiles by pie chart

(A) Comparative analysis showed that when the CG methylation was up-regulated, there were 72 genes up-regulated and 44 genes down-regulated at transcription level, while 924 genes remained stable.

(B) When the CG methylation was down-regulated, the number of up-regulated, down-regulated and unchanged genes at transcription level were 406, 182 and 3559, respectively.

Supplementary table 3. The CpG islands sequences in three ICC marker genes

Gene Symbol	The CpG islands sequences	chromosome	Integration loci
EHF 1#	cgttaaacatcaacagattaaactggcaggattgctgaagcgtgtgtgtgtgtgcttcatagtgaaagtagctctctacaatcctttttttttttttggcatcatgcaaatacactcacctggcatgaatatcagccaatcaaagcagaaggaagcaggtagaataaaaatcctcattggtcagcgtgaagttcataagttcagcaggcctaaacactgacaggttcacagttctgtctctctatcaaactcaaacctctgtcccgtacagacaccaaacacttcagacaactttaactttctagtccg	25	10319582-10319891<br>10319582-10319777: Upstream sequence<br>10319778-10319891: 5'UTR
EHF 2#	cgtaaccaaatattttaatattgttaataaacacaaattaatggaataatttcatcgaaataactttagcgtgagagcatcaaaacattgagttaatctgtctagtttttttttttttttaggaaaaatacacaagcttctaatatcctttgtttgcttgaggtgtgaacattatctgttgttagcatagctcctgtcggttttacacacattccataaaatgatctcctaggccatctcagaatcaataaaaaa	25	10324905-10325159<br>(Intron 1)
EPHA4	cgtaaatcgagtttgcttttcatttccaaaaggcagacgctgcctaattgcctgtcttccagttataagtggtatatttatgttctgagcaaatatcaggggctccggggttaaagcccggttcagtatctacttggaagaacagagtagcggtttaagtgagagtaatgcattcaacattgggctgcactttgacctctgtgttcg	2	40624668-40624874<br>(Intron 11)
ITGB6	cgtgaggcaaacaaaaagcaatgtggttttttttttctctgtgtgtgatagaataagaaatatcagtgtgtgaatcacttgtgtattcagctctccatataacatgttcaaacacagccttgatcacatcacccagaccgacctctcctgacacaaatatttcatgttttctgagctctccttctcttgctctttatcactcgcgttcatctcagattctgtggctgttctccagtttagagttgatgtttaacca	11	11011880-11012134<br>(Intron 7)

#Different CpG islands in *EHF*. The hypomethylated sites in these CpG islands are indicated in grey.

Supplementary table 4. RT-qPCR primers

Gene Symbol	Forward Primer (5'To 3')	Reverse Primer (5'To 3')
AGR2	TGTCAGTGCTCTTGGTCATG	CTTCCTCGTATGTCTGTGCC
ECE2	AGACGCTGGGAGAAAACATC	AAGAGCTGGTCGTTTGTCAG
EHF	TTCCCGTGTTCAATTCTCCC	CTTTTGACTTGTGACCGCTTG
EPCAM	TTTGGATAAGAAACTTGTGTCTGAAG	TGTAGTACGGTCGTCCTTATCTTTTT
EPHA4	CACACCAACTACACCTTCCA	GCGTGATGTCCTTACTCTGAA
EPS8L3	TGCCAGTCCACCGATTAAAG	TGTGGAGGTGGAAAATCTGG
ITGB6	CGGTGGAGATAAAAGGCTGTC	TGTTGGTTTCGGGTGTCTG
PDZK1IP1	CCTCTTTCTCGTCTTCATCTCC	ATTCGGTATGACTGCTTCTGG
SLC44A4	ACATACAAGCCAACCAGACC	AAGCCCTCGGTGTTATAGTTG
SLC6A14	AGCTACTTTCCCTTACATCGTG	TCTTTCCAAACCTCAGCCTC
SPINT2	ATAACTTTTACTCCCAGGCGG	TCATTTCAGTAAGAGCCTTGGG

Supplementary table 5. Methylation PCR primers

Gene Symbol	Forward Primer (5'To 3')	Reverse Primer (5'To 3')
EHF 1#	GGTAAATAGGATGGTTTAG	TCCCCAATAAAAAATCAAAAAA
EHF 2#	TGTTTTATTTAAAGTTTACATT	TAACAACATATATAAACAAC
EPHA4	GACCTTTTGTTAGCATTGG	TCTCCCACTAAACCTTCTG
ITGB6	GTTGTGATTAAAGATTAGAAGG	AAAATCAAATACCAAAACCCCCA

#Different CpG islands in *EHF*.

Full-length blots/gels of Supplementary Figure1 are presented

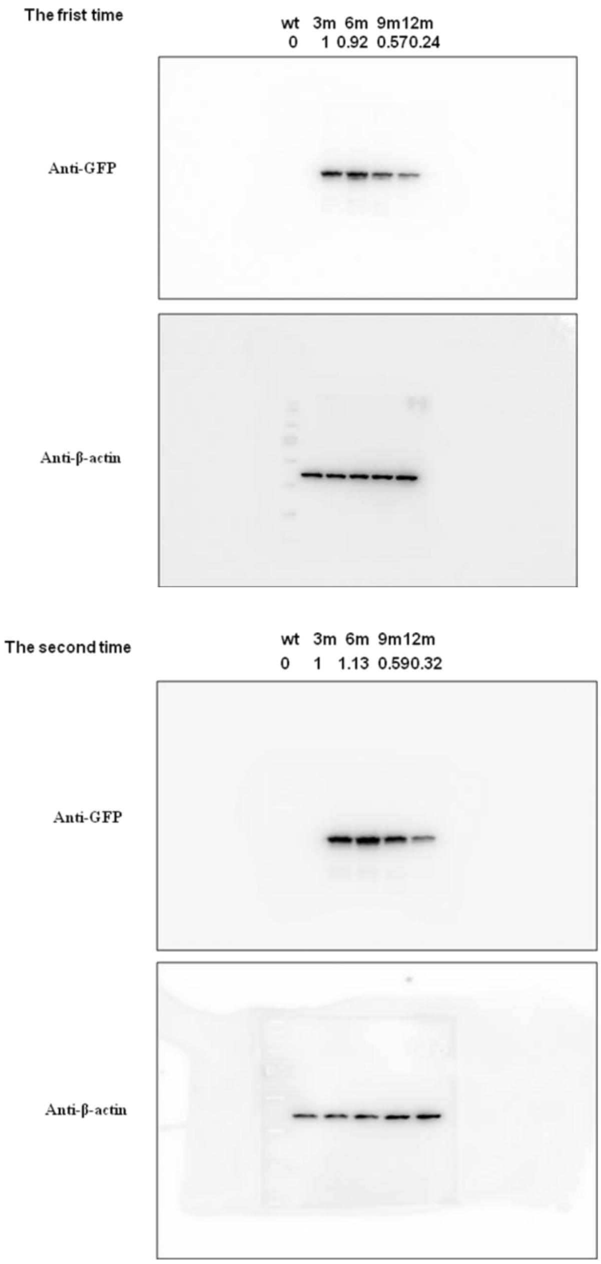

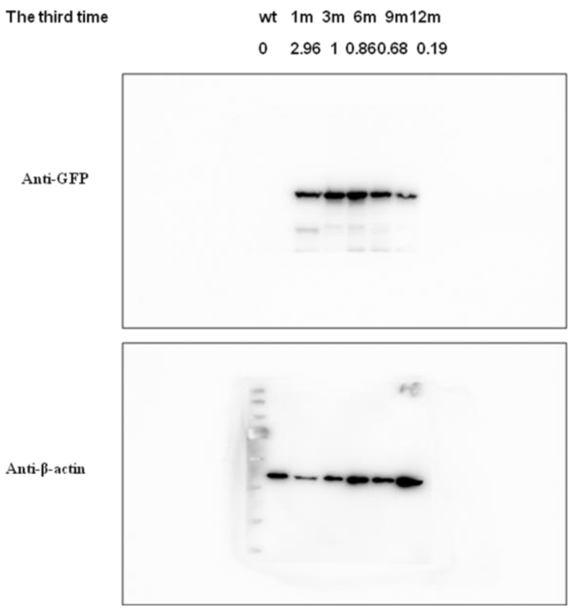

Full-length blots/gels of Supplementary Figure2 are presented

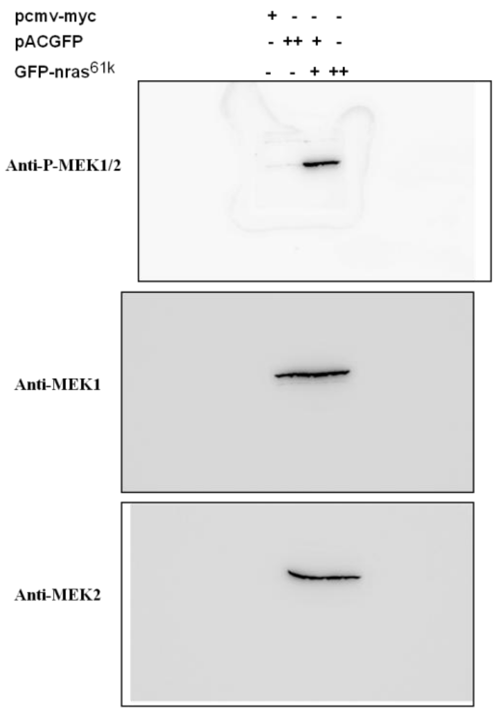

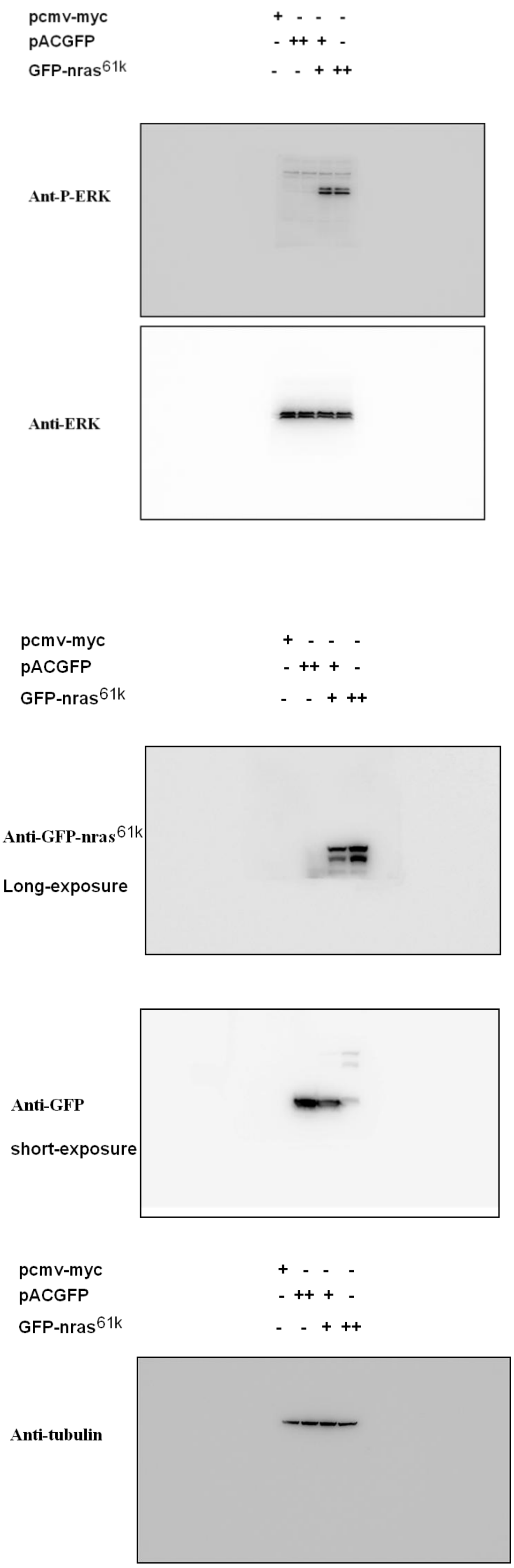

pcmv-myc + - - -
pACGFP - ++ + -
GFP-nras^{61k} - - + ++
Ant-P-ERK
Anti-ERK
pcmv-myc + - - -
pACGFP - ++ + -
GFP-nras^{61k} - - + ++
Anti-GFP-nras^{61k}
Long-exposure
Anti-GFP
short-exposure
pcmv-myc + - - -
pACGFP - ++ + -
GFP-nras^{61k} - - + ++
Anti-tubulin
